# CAR T-cell therapy in multiple myeloma: more room for improvement

**DOI:** 10.1038/s41408-021-00469-5

**Published:** 2021-04-29

**Authors:** Phaik Ju Teoh, Wee Joo Chng

**Affiliations:** 1 Department of Medicine, Yong Loo Lin School of Medicine, National University of Singapore, Singapore, Singapore; 2 Cancer Science Institute of Singapore, Singapore, Singapore; 3 Department of Haematology-Oncology, National University Cancer Institute of Singapore, National University Health System, Singapore, Singapore

**Keywords:** Cancer immunotherapy, Translational research, Myeloma

## Abstract

The emergence of various novel therapies over the last decade has changed the therapeutic landscape for multiple myeloma. While the clinical outcomes have improved significantly, the disease remains incurable, typically in patients with relapsed and refractory disease. Chimeric antigen receptor (CAR) T-cell therapies have achieved remarkable clinical success in B-cell malignancies. This scope of research has more recently been extended to the field of myeloma. While B-cell maturation antigen (BCMA) is currently the most well-studied CAR T antigen target in this disease, many other antigens are also undergoing intensive investigations. Some studies have shown encouraging results, whereas some others have demonstrated unfavorable results due to reasons such as toxicity and lack of clinical efficacy. Herein, we provide an overview of CAR T-cell therapies in myeloma, highlighted what has been achieved over the past decade, including the latest updates from ASH 2020 and discussed some of the challenges faced. Considering the current hits and misses of CAR T therapies, we provide a comprehensive analysis on the current manufacturing technologies, and deliberate on the future of CAR T-cell domain in MM.

## Introduction

Multiple myeloma (MM), which accounts for 10% of blood cancers, is a malignancy of plasma cells originating in the bone marrow. Increased understanding of disease biology over the years has led to parallel improvement in treatment modalities, evidenced by the emergence of the novel therapeutics such as proteasome inhibitors (bortezomib, carfilzomib, and ixazomib), immunomodulatory drugs (thalidomide, lenalidomide, and pomalidomide) and U.S. Food and Drug Administration (FDA)-approved monoclonal antibodies (daratumumab and elotuzumab)^1–4^. While these new therapeutics have led to better disease control, myeloma remains largely incurable, and high-risk patients do not benefit much from this armamentarium of therapies^4–7^. Drug resistance is inevitable and disease relapse remains a great clinical challenge.

Immunotherapy, which was previously deemed a favorable concept, has now evolved into a practical cancer treatment and its progress in the past decade has revolutionized the cancer therapy landscape^8,9^. Chimeric antigen receptor (CAR) T-cell therapy is one of the rapidly emerging and highly promising immunotherapeutic options that has shown unprecedented results in B-cell malignancies^10–13^. It prolongs patients’ survival and remission, even for some whom standard treatments have failed^14^.

**Fig. 1:**
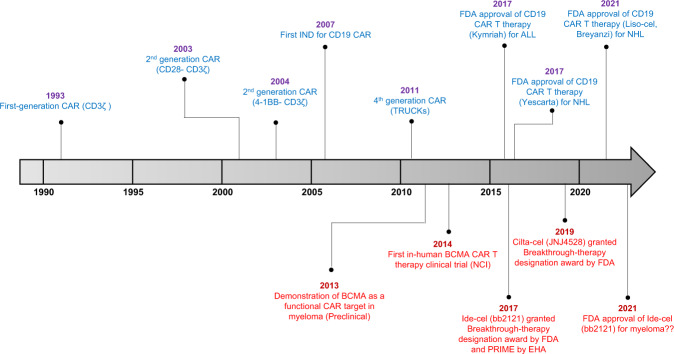
The time line of CAR T-cell development. The history of CAR T cells and its progress and milestones achieved over the years. Red fonts are the time line for CAR T-cell development in myeloma.

The success of CAR T-cells therapy in cancer is exemplified by the FDA-approved anti-CD19 CAR T-cell products, namely, tisagenelcleucel/Kymriah, (Novartis) for the treatment of acute lymphoblastic leukemia (ALL) and axicabtagene ciloleucel/Yescarta, (Gilead/Kite) for diffuse large B-cell lymphoma (DLBCL). Most recently (February 2021), CAR T therapy scene hits another milestone when liso-cel/Breyanzi (Juno/BMS) has also received the FDA-approval for DLBCL treatment, owing to its remarkable efficacy and low incidence of high-grade toxicity^15,16^ (Table 1 and Fig. 1).

**Table 1:** FDA-approved anti-CD19 CAR T-cell therapies (true to the time of writing).

	Tisa-cel (Kymriah)	Axi-cel (Yescarta)	Liso-cel (Breyanzi)
Study	JULIET	ZUMA-1	TRANSCEND NHL 001
Trial number	NCT02445248	NCT02348216	NCT02631044
*N* (evaluable)	111	101	255
Ectodomain	Anti-CD19	Anti-CD19	Anti-CD19
Origin of antibody	Murine FMC63 scFv	Murine FMC63 scFv	Murine FMC63 scFv
Hinge/Transmembrane	CD8	CD28	IgG4
Endodomain	4-1BB-CD3ζ	CD28-CD3ζ	4-1BB-CD3ζ + eGFRt
Genetic manipulation	Lentivirus	Retrovirus	Lentivirus
Starting cells for manufacturing	Bulk T cells	Bulk T cells	CD4+/CD8+
Clinical efficacy	54% (CR 40%)	83% (CR 58%)	73% (CR 53%)
Toxicity	CRS: 68% Neurotoxicity: 14%	CRS: 94% Neurotoxicity: 31%	CRS: 42% Neurotoxicity: 30%
Survival outcome	Median PFS: 3 months Median OS: 8 months	Median PFS: 8.3 months Median OS: NA	Median PFS: 6.8 months Median OS: 21.1 months

These unprecedented results in B-cell malignancies have spurred a tidal wave of CAR T research in other cancers, including MM. This review details the general development of CAR T-cell therapy and its role in paradigm shift for myeloma treatment. Our topics of discussion range from the current state of its clinical development to the latest technologies adopted in CAR manufacturing for myeloma. Here, we also deliberated on the future perspectives and proposed ways to potentially improve the CAR T-cell research, as we maneuver towards revolutionizing the CAR T scene in myeloma.

## Overview of CAR T-cell therapy

The history of CAR T-cell therapy dates back to more than two decades ago, when the first ever CAR T cells were created against 2,4,6-trinitrophenyl (Fig. 1). Although an activated immune response was observed, systemic persistence was unfortunately lacking^17^. Subsequent research remained lacklustre until another decade later, the second-generation CAR T cells directed against CD19 emerged with proven efficacy in preclinical B-cell models^18^. This sets the precedence for the promising venture of CD19-CAR T therapy in ALL and chronic lymphocytic leukemia (CLL)^19^.

**Fig. 2:**
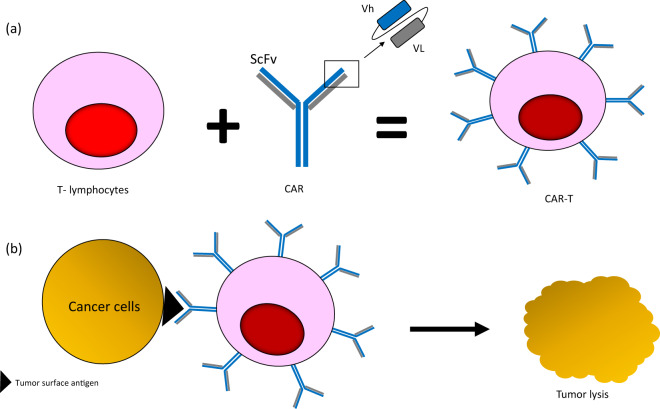
The basic principle of CAR T-cell therapy. **a** T lymphocytes are genetically modified to express chimeric antigen receptor which is made up of monoclonal antibody targeting specific antigen of interest. ScFV Single chain variable fragment, Vh Heavy chain, VL Light chain. **b** ScFV portion of CAR T cell recognizes tumor-associated antigen on the surface of tumor cells, binds to them, and initiates a cascade of cytotoxic signaling, leading to tumor lysis.

CAR T-cell therapy is able to reprogram host’s immune system to attack tumor cells without the physiological need for HLA-presentation. T lymphocytes are biologically engineered to express monoclonal antibodies (moAb) recognizing tumor-associated antigens (Fig. 2). Engagement of these cognate antigens by the antibody-bound T cells will initiate signaling cascades within the T cells that stimulate release of pro-inflammatory cytokines such as TNF-α, IFN-γ, IL2, and IL6, leading to cytolysis^20^. This unique CAR T-cell property can help mitigate the common limitations of T-cell receptor (TCR)-induced immunity, including the Major Histocompatibility Complex (MHC) loss on tumor cells and low antigen-binding affinity of T cells^21–23^.

**Fig. 3:**
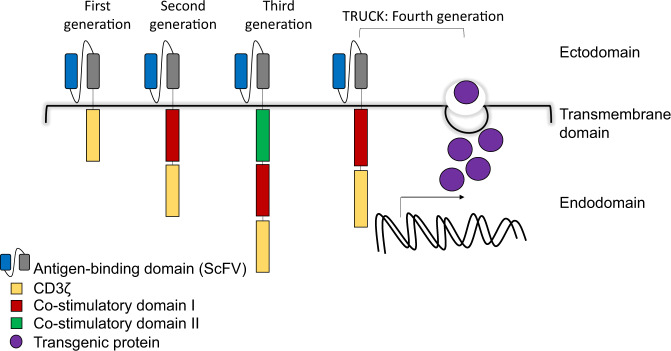
The depiction of different generations of CAR T cell. First-generation CAR contains basic design with the ScFV portion of monoclonal antibody at the ectodomain, hinge at the transmembrane domain and CD3ζ signaling molecule at the endodomain. Second- and third-generation CARs comprise additional one or two co-stimulatory molecule/s in the endodomain, respectively. Fourth-generation CAR (TRUCK) follows the basic design of second-generation CAR, coupled with an added transgene encoding for a cytokine (transgenic protein).

Figure 3 depicts the basic components of a CAR. The single chain variable fragment (ScFV) of a monoclonal antibody on the ectodomain harbors antigen recognition function and is linked to the intracellular domains by a hinge/transmembrane region, commonly derived from CD8 or IgG4. The first-generation CAR T cell contains only the CD3ζ signaling domain, which lacks proliferation profile^17^. The current and conventionally produced CAR T products incorporate either one (second-generation) or two (third-generation) co-stimulatory domains (4-1BB, CD28, and/or OX-40) to promote efficient CAR T-cell signaling, persistence, and efficacy^24–27^. The more sophisticated fourth-generation CAR, T-cell redirected for universal cytokine-mediated killing (TRUCK), consists of an added transgene encoding for a pro-inflammatory cytokine, which when induced by signaling molecules, is released to mediate cytotoxicity^21,28,29^.

**Fig. 4:**
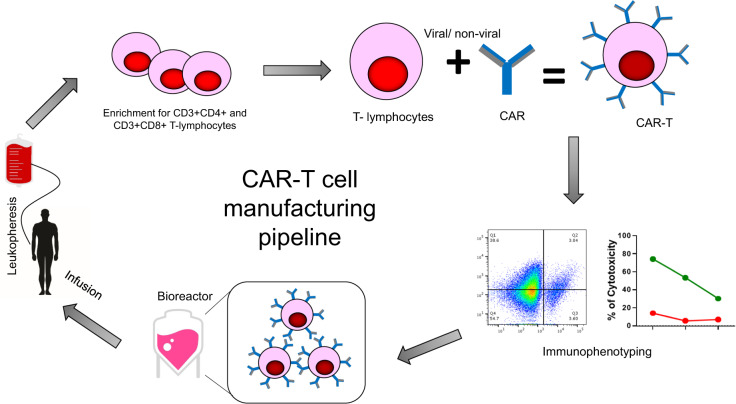
CAR T-cell manufacturing pipeline. The process starts off with leukapheresis to obtain PBMCs from the patients. Enrichment of T cells is done prior to genetic modifications via viral or non-viral gene delivery system. This is followed by immunophenotyping analysis to determine the cytokine and cytolytic profile of the CAR T cells. Positive CAR T cells are selected to undergo ex vivo expansion in a bioreactor vessel containing growth factor-enriched media. When the required amount is obtained, the CAR T cells will be transported back to the clinic for patient infusion.

CAR T-cell manufacturing is a laborious process which takes 2–4 weeks for completion in a Good Manufacturing Practice (GMP) setting (Fig. 4). CAR T-cell therapy is predominantly autologous, and the manufacturing process starts with the collection of peripheral blood mononuclear cells (PBMCs) from patients via leukapheresis. T-cell population is enriched prior to genetic modification with CARs of interest. Gene delivery is performed via viral transduction (γ-retrovirus or lentivirus) or non-viral transfer technology (DNA transposon or mRNA transfection)^30^. This is followed by immunophenotyping analyses to ensure successful endowment of T cells with CARs and cytolytic activities. These CAR T cells will then undergo ex vivo expansion within a bioreactor, containing growth factor-enriched media, before finally being frozen for storage or immediately transported to the clinic for infusion.

## CAR T-cell therapy in MM

CAR T research in myeloma is still at its infancy, comparatively to lymphoma and ALL (Fig. 1). The most widely studied myeloma CAR target is none other than the B-cell maturation antigen (BCMA), a tumor necrosis factor receptor superfamily member 17 (TNFRSF17). It is deemed an ideal antigenic target due to its preferential expression on the plasma cells but not on hematopoietic stem cells^31^. Binding and stimulation of ligands, B-cell Activating Factor (BAFF) and A Proliferation-Inducing Ligand (APRIL), to BCMA promotes growth and proliferation of plasma cells in the bone marrow. Although the expression of BCMA is heterogenous^32,33^, it is universally present in all MM cells^31,34^ and its overexpression carries important prognostic value^35,36^.

The first BCMA-directed CAR was developed less than a decade ago, showing preclinical evidence of functional targetability^31^. This was followed by the first-in-human Phase I clinical trial to test the efficacy of the BCMA-targeted CAR T cells in relapsed/refractory multiple myeloma (RRMM) (NCT02215967). The overall response rate (ORR) was a good 81%, with 63% demonstrating very good partial (VGPR) or complete response (CR)^37,38^. Consequentially, there was a widespread effort to develop novel anti-BCMA CARs and to fine-tune the old CARs.

Table 2 documents the anti-BCMA CAR T-cell clinical trial records, with the latest updates from ASH 2020^38–40^. The first significant breakthrough in CAR T-cell development for myeloma was reached when bb2121 (Bluebird Bio) was granted a breakthrough-therapy designation award by the FDA and Priority Medicine (PRIME) eligibility award by the European Medicines Agency (EMA) in 2017. This was precipitated by its excellent efficacy and durable responses. Latest reported ORR was 73% with significantly improved PFS (median PFS 8.8 months) in the RRMM patients^41,42^. Bb2121 has been heralded as the latest front-runner for FDA’s approval for MM treatment. Its successor bb21217, designed with the same CAR construct but is being cultured in the presence of PI3K inhibitor bb007 ex vivo, has recorded a decent ORR of 55%, associated with enrichment of memory T-cell phenotype and high-peak expansion^43^. Additionally, JNJ-4528, the bi-epitope CAR T-cell product containing two BCMA-targeting domains, aimed to enhance the antigen binding affinity, has also shown an impressive 94.8% ORR in the CARTITUDE-1 trial^44,45^. This study provides compelling evidence that a single low-dose infusion of the CAR T product was sufficient to induce early, deep and durable response. JNJ-4528 takes after the exact construct of LCAR-B38M that is being evaluated in another geographical cohort (China) in the LEGEND-2 trial. The study reported a promising ORR of 82.4% with a good safety profile in the high-risk patient subgroup^46^.

**Table 2:** Anti-BCMA CAR T-cell clinical trials in multiple myeloma (true to the time of writing).

Trial number (name)	Site/ Sponsor	CAR	ScFV origin	Phase	*n* (at the time of reporting)	Antigen	Co-signaling domain/ CD3z	Design	Transfer method	Cell source	Conditioning	Dosage (CAR T cells/kg)	BCMA expression	Responses	Toxicity	Survival outcome	Additional info
NCT02215967	NCI	CAR-BCMA	Murine	1	16	BCMA	CD28	2nd generation	Retroviral	Autologous	CP/Flu	0.3–9.0 × 10^6^	>50%	ORR: 81%(2 sCR, 8 VGPR, 3 PR)	CRS: 15 (93.75%), including 2 Grade 4, 4 Grade 3, 7 Grade 2, and 2 Grade 1	Median PFS: 7.2 months	Culture medium: anti-CD3 MoAb and IL2
NCT03361748 (KarMMa)	Bluebird bio	bb2121/ Ide-cel	Murine	1	128	BCMA	4-1BB	2nd generation	Lentiviral	Autologous	CP/Flu	150–450 × 10^6^	>50%	ORR: 73% (33% CR) MRD (<10 − 5 nucleated cells): 26%	1. CRS: 84%, 5% Grade ≥3 2. Neurotoxicity: 18%, 3% Grade ≥3	Median PFS: 8.8 months	Culture medium: anti-CD3/CD28, OKT3
NCT03274219 (CRB-402)	Bluebird Bio	bb21217	Murine	1	46	BCMA	4-1BB + PI3K inhibitor domain	2nd generation	Lentiviral	Autologous	Flu/CP	15, 30, 45 × 10^7^	>50%	ORR: 24 (55%), 8 (18%) with ≥CR and 13 (30%) with VGPR Median DOR: 11.9 months	1. CRS: 67%, including 14 Grade 1, 15 Grade 2, 1 Grade 3 and 1 Grade 5 2. 22% neurotoxicity, including 5 Grade 1, 2 Grade 2, 2 Grade 3, 1 Grade 4	NA	1. Culture medium: additional PI3K inhibitor bb007 2. Enrichment for memory like T cells in bb21217 3. Presence of early memory-like T cells is associated with high peak expansion and prolonged response
NCT03090659 (LEGEND-2)	Nanjing Legend Biotech Co	LCAR-B38M	Llama	1/2	57	Biepitope to BCMA (VHH1 and VHH2)	4-1BB	2nd generation	Lentiviral	Autologous	CP	0.5–5 × 10^6^	Yes (cut-off not reported)	ORR for EMD: 82.4% ORR for non-EMD: 90%	1. CRS: 90%, Grade 1 (47%), Grade 2 (35%); Grade 3 (7%) 2. Neurotoxicity: 1 (Grade 1), dosed at 1.0 10^6^ cells/kg	Median follow up: 25 months Median PFS in EMD: 8.1 months Median PFS in non-EMD: 25 months	Bispecific anti-BCMA variable fragments of llama heavy-chain antibodies
NCT03090660	Nanjing Legend Biotech Co	LCAR-B38M	Murine	1	17	Biepitope to BCMA (VHH1 and VHH2)	4-1BB	2nd generation	Lentiviral	Autologous	CP	0.21–1.52 × 10^6^	Yes (cut-off not reported)	ORR: 88.2% (13 sCR, 2 VGPR);	1. CRS:100%, including 10 Grade 1/2, 6 Grade 3, and 1 Grade 5. 2. No neurotoxicity	12-months PFS: 52.9%	Bispecific anti-BCMA variable fragments of llama heavy-chain antibodies
NCT03548207 (CARTITUDE-1)	Johnson & Johnson	JNJ-4528/ cilta-cel	Llama	1/2	25	Biepitope to BCMA (VHH1 and VHH2)	4-1BB	2nd generation	Lentiviral	Autologous	NA	0.75×10^6^	Yes	ORR: 94.8% (sCR-55.7%, VGPR-32%, PR-7.2%) 6 months PFS: 87.4% 6 months OS: 93.8%	CRS: 94.8%, (49.5% Grade 1, 39.2% Grade 2, 4.1% Grade 3, and 1% Grade 5) Neurotoxicity: 20.6% (10.3% Grade 3/4)	NA	1. CAR design takes after LCARB38M; Culture medium: IL-2 2. 67% patients had CAR T-cells below the level of quantification (2 cells/µL) in peripheral blood
NCT03430011 (EVOLVE)	Juno Therapeutics, Inc	JCARH125 /Orva-cel	Human	1/2	115	BCMA	4-1BB	2nd generation	Lentiviral	Autologous	CP/Flu	50, 150, 300, 450 and 600 × 10^6^	NA	ORR: 82% (27% CR)	1. CRS: 75%, all Grade 1or 2 2. Neurotoxicity: 3 (2 Grade 1, 1 Grade 3)	NA	1. 1:1 CD4/CD8 ratio preselected prior to transduction and expansion. 2. Response did not correlate with baseline serum BCMA level 3. Serum BCMA declined with treatment, more so in responders.
NCT03975907 (LUMMICAR-1) China	CARsgen Therapeutics	CT053	Human	1	24	BCMA	4-1BB	2nd generation	Lentiviral	Autologous	CP/Flu	1.0-1.5×108	Yes (no cut-off reported)	ORR: 100% (4 sCR, 1 CR, 3 VGPR and 4 PR) All 5 subjects with CR/sCR were minimal residual disease (MRD)-negative at the 10^5^ sensitivity level.	CRS: 91.7%, (8 Grade 1 and 3 Grade 2)	NA	Peak expansion at 7–14 days after dosing in all subjects
NCT03915184 (LUMMICAR-2) North America	CARsgen Therapeutics	CT053	Human	1/2	20 enrolled, 14 received treatment	BCMA	4-1BB	2nd generation	Lentiviral	Autologous	CP/Flu	8 patients received 1.5–1.8 × 10^8^ 6 patients received 2.5–3.0 × 10^8^	Yes (no cut-off reported)	ORR: 100% (2 sCR, 2 CR, 1 VGPR and 5 PR) 11 were MRD-negative at the 10^5^ sensitivity level	1. CRS: 86% Grade 1 or 2 2. Neurotoxicity: 1 subject Grade 2	NA	NA
NCT03070327	Poseida Therapeutics, Inc	MCARH171	Human	1	11	BCMA	4-1BB + EGFRt	2nd generation	Retroviral	Autologous	CP/Flu	72–818 × 10^6^	>1%	1. ORR:64% 2. 100% ORR observed in 5 patients received higher doses (450 × 10^6^)	1. CRS: 6 (60%), 4 grade 1–2, and 2 grade 3. 2. No grade 3 neurotoxicity	NA	No predefined CD4/CD8 ratio. Higher doses correlated with peak expansion and durability of response.
ChiCTR-OPC-16009113	Huazong University	BCMA-CAR T cells	Murine	1	28	BCMA	CD28	2nd generation	Lentiviral	Autologous	CP/Flu	5.4–25.0 × 10^6^	Yes (no cut-off reported)	Strong BCMA expression (*n* = 22): ORR, 87% (73% CR); Weak BCMA expression (n = 6): ORR, 100% (33% CR or VGPR)	14% grade 3CRS.	Median DFS (strong vs weak): 296 vs 64 days	NA
NCT03661554	ShenZhen Pregene Biotechnology Company, Ltd	BCMA-CAR T cells	Humanized alpaca	1	16	BCMA	4-1BB	2nd generation	Lentiviral	Autologous	CP/Flu	2–10 × 10^6^	Yes (no cut-off reported)	8 ORR: 84.6% (28 days, 13 patients) ORR: 100% (3sCR/CR, 1 VGPR, 3 PR) (10 weeks, 7 patients)	2 patients with grade 3–4 CRS (0–2 in other patients)	NA	NA
NCT02546167	University of Pennsylvania–Novartis Alliance	CART-BCMA	Human	1	25	BCMA	4-1BB	2nd generation	Lentiviral	Autologous	With or without lymphdepletion	Cohort 1: 1–5 × 10^8^ CART-BCMA cells Cohort 2: CP 1.5 g/m2 + 1–5 × 10^7^ CART-BCMA cells Cohort 3: CP 1.5 g/m2 + 1–5 × 10^8^ CART-BCMA cells	Not required	ORR: 48% (CR- 8%) Cohort 3: ORR, 64%; CR: 9%	1. CRS: 22 (88%); 8 grade 3–4 (all 1–5 10^8^ dose) 2. Neurotoxicity (n = 8, 32%): 5 grade 1–2, 3 grade 3–4	Median PFS: Cohort 1- 2.2 months, Cohort 2- 1.9 months, Cohort 3- 4.2 months OS: 502 days	BCMA intensity did not predict response. Responses correlated with CAR T-cell expansion with higher proportion of naive/memory phenotype cells. BCMA declined with treatment, more so in responders.
NCT03093168	Henan University (HRAIN)	Anti-BCMA CAR	Human	1	46	BCMA	4-1BB + EGFRt	2nd generation	Retroviral	Autologous	CP/Flu	9 × 10^6^	>5%	ORR: 79.6% (2sCRs, 16CRs, 8VGPRs and 8PRs) MRD negativity: 16 patients	CRS: 22.7% Grade 1-2, 6.8% Grade 3 CRS. No grade 4 CRS reactions developed and all toxicities were fully reversible. Neurotoxicity: 1(7%)	Median PFS: 15 months 24-months PFS: 49.16% 24-months OS: 53.95%	NA
NCT03338972	Fred Hutchinson Cancer Research Center	FCARH143	Human	1	11	BCMA	4-1BB + EGFRt	2nd generation	Lentiviral	Autologous	CP/Flu	5 and 15 × 10^7^	>5%	ORR: 100% (sCR/CR: 55%, VGPR: 36%, PR: 9%)	1. CRS: 91% (Grade 1 and 2) 2. Neurotoxicity: 9%	NA	Infusion of 1:1 CD4/CD8 CAR-T ratio. BCMA antigen loss seen in 1 patient at time. CAR T cells remained detectable 90 days post infusion, representing ≤41.5% of CD3 + lymphocytes of relapse.
NCT03288493	Poseida Therapeutics, Inc.	P-BCMA-101 (CARTyrin)	Human	1, 3 + 3	43	BCMA	4-1BB + safety switch	2nd generation	Piggyback-transposon	Autologous	CP/Flu	0.75–15 × 10^6^	Not required	ORR: 100% (single CAR T agent or in combination with rituximab and lenalidomide)	1. CRS:17% (1 Grade 3) 2. Neurotoxicity: 1 Grade 2	NA	1. Nonviral PiggyBac DNA-delivery system: increases memory stem cells to increase persistence. It has a proprietary safety switch. Peak expansion was delayed/slower (14–21 days) 2. Addition of rituximab or lenalidomide pre- and post- lymphodepletion to prevent anti-CAR antibody development and increase T cell robustness, respectively
NCT03602612	National Cancer Institute (NCI)	FHVH33	Human	1	15	BCMA	4-1BB	2nd generation	Retroviral	Autologous	CP/Flu	0.75 and 12.0 × 10^6^	Yes	sCR/CR (20%) VGPR (7%) PR (53%)	1. CRS Grade 1–2 (87%), Grade≥ 3 (7%) 2.Neurotoxicity (27%)	NA	NA
NCT03549442	Abramson Cancer Center	CAR-T-BCMA (Upenn) + CTL119 (humanized tisagenlecleucel)	Human	1	A = 6; B = 4	CD19 + BCMA	4-1BB	2nd generation	Lentiviral	Autologous	CP/Flu	5 × 10^8^ total CAR-T	Yes (cut-off not reported)	ORR: 80% (1CR, 4VGPR, 3PR)	CRS:80%, all grade1-2	NA	NA
NCT03455972	The First Affiliated Hospital of Soochow University	CART-19/BCMA (cocktail)	NA	1/2	9	CD19 + BCMA	OX40/CD28 + EGFRt	3rd generation	Lentiviral	Autologous	NA	1 × 10^7^ (CAR-T-CD19); Split dose for CART-BCMA (40% d1, 60% d2)	All with BCMA > 50% without CD19 expression on PCs	ORR: 100% (3 CR, 6 VGPR)	1. CRS: 100%, all Grade1–2; no serious neurotoxicity	NA	Culture medium: Anti-CD3 beads
NCT03778346	The Sixth Affiliated Hospital of Wenzhou Medical University	NA	NA	1	30 (estimated)	Integrin β7, BCMA, CS1, CD38 and CD13 (single or up to 10 combination)	NA + simultaneously expressing IL7 and CCL19	4th generation	NA	Autologous	NA	10^6^—10^7^ Total CAR-T	NA	NA	NA	NA	NA
NCT03271632	Shenzhen Geno-Immune Medical Institute	NA	NA	1/2	20 (estimated)	BCMA, CD38, CD56, CD138	NA	4th generation	NA	Autologous	NA	NA	Yes (cut-off not reported)	NA	NA	NA	To investigate the persistence and function of CAR T cells in the body after CAR T-cell infusion
NCT03196414	The First Affiliated Hospital of Soochow University	SZ-MM-CART01	NA	1	29	BCMA, CD19, CD138	OX40/CD28	3rd generation	Lentiviral	Auto/allo	CP/Flu	20–82 × 10^6^ total CAR-T	NA	ORR:100% (sCR/CR: 54%, VGPR: 4%, PR: 29%)	1. CRS: 66% Grade 1 and 2, 34% ≥ Grade 3 2. Neurotoxicity: 3%	NA	NA
NCT03287804	Autolus Limited	AUTO2	NA	1/2	12	Ligand based: APRIL (BCMA and TACI)	CD28/OX40 + RQR8 safety switch	3rd generation	Retroviral	Autologous	CP/Flu	15–900 × 10^6^	NA	ORR: 43% (28% PRs and 14% VGPRs)	45% CRS, all grade 1, no neurotoxocity	NA	Preliminary efficacy seen to date following treatment has been determined not sufficient to warrant further development- trial is terminated
NCT03767751	Chinese PLA General Hospital	NA	NA	1/2	80	Bispecific to CD38 + BCMA	NA	NA	NA	Autologous	NA	1–5 × 10^6^	NA	NA	NA	NA	NA
NCT03473496	Zhujiang Hospital	NA	NA	1/2	50	CD38, BCMA, CD138, CD56 (single or double combination)	NA	NA	NA	Autologous	NA	10^6^–10^7^	NA	NA	NA	NA	NA
NCT03271632	Shenzhen Geno-Immune Medical Institute	NA	NA	1/2	20	CD38, BCMA, CD56 (single or multi)	NA	NA	NA	Autologous	NA	NA	NA	NA	NA	NA	NA
NCT03638206	Shenzhen BinDeBio Ltd.	NA	NA	1/2	73	CD38, BCMA, NY-ESO-1 (single or multi)	NA	NA	NA	Autologous	CP/Flu	NA	Yes (no cut-off reported)	NA	NA	NA	If NY-ESO-1 is positive expression, positive HLA-A*0201 is required at the same time
NA	National Cancer Institute, Bethesda	FHVH33	Human	1	21	BCMA	4-1BB	2nd generation	Retroviral	Autologous	CP/Flu	0.75 × 10^6^, 1.5 × 10^6^, 3 × 10^6^, 6 × 10^6^ and 12 × 10^6^	NA	ORR: 90% (12 sCR, CR and VGPR)	1. CRS: 95% (76% Grade 1 or 2, 19% Grade 3) 2. Neurotoxicity: 38% (23% Grade 1 or 2, 9% Grade 3, 4% Grade 4)	NA	NA
NCT04236011 NCT04182581	Shanghai Changzheng Hospital; Gracell Biotechnologies (Shanghai) Co., Ltd	GC012F	NA	Early 1	16	BCMA and CD19 (manufactured with FasT CARplatform)	NA	NA	Lenviral	Autologous	CP/Flu	Dose Level 1: 1 × 10^5^/Kg (DL1) (1 patients) Dose Level 2: 2 × 10^5^/Kg (DL2) (9 patients) Dose Level 3: 3 × 10^5^/Kg (DL3) (6 patients)	Clear expression by flow	ORR: 93.8% (sCR/CR: 56.3%) MRD negative: 8.6% (11/14) at month 1, 100% at month 3 (11/11) and 100% at month 6 (10/10)	CRS Grade 1–2: 87.5% CRS Grade 3: 12.5%	NA	The CAR-T median proliferation peak was reached on Day10 (Day8–Day14)
NCT04155749	Arcellx, Inc	ARC-101	Synthetic (non-scFv)	1	3	BCMA	4-1BB	2nd generation	NA	Autologous	CP/Flu	100, 300, and 900 × 10^6^ cells	Not required	1 sCR (MRD-10-4), 1 sCR, 1 sCR (MRD-10-6)	1. CRS: 3 subjects 2. Neurotoxicity: 1 subject	NA	Median drug product CAR T expression was 76% (min:max 72–78%) of total CD3 + T cells
NCT04322292 NCT03815383 NCT03751293 NCT04295018	Institute of Hematology & Blood Diseases Hospital	C-CAR088	Human	1	21	BCMA	4-1BB	2nd generation	Lenviral	Autologous	CP/Flu	1.0 × 10^6^ cells (*n* = 3), 3 × 10^6^ cells (*n* = 11) and 4.5~6 × 10^6^ cells (*n* = 7)	NA	Best overall response included 6 complete responses (CRs), 10 very good partial responses (VGPRs) and 4 partial responses (PRs) In the 3 × 10^6^ CAR-T cells/kg dose group, 5/11(45%) patients achieved CR	CRS: 20 patients (95%) Grade 1–2, 1 patient Grade 3	NA	1. Cultured in serum-free, automated and digital, closed system 2. The median vein to vein time was 16 days 3. Median time to CRS was 6.5 days (range: 1–11 days) and median duration of CRS was 5 days (range: 2–10 days)
NCT04093596 (UNIVERSAL)	Allogene Therapeutics	ALLO-715	NA	1	19 (estimated)	BCMA	NA	NA	NA	Allogeneic	CP or Fu or ALLO-647 (anti-CD52 mAb, for selective and prolonged host lymphodepletion)	40, 160, 320, and 480 × 10^6^	NA	ORR for DL3 (320 × 10^6^ CAR T): 60% (1 sCR and 1 VGPR)	CRS: 4 pts (24%). Three episodes were Grade 1 and 1 was Grade 2; all resolved without tocilizumab or corticosteroids	NA	All responses were initially observed at day 14
NCT04613557 (IMMUNICY-1)	Celyad Oncology SA	CYAD-211	NA	1	12 (estimated)	BCMA	NA	NA	NA	Allogeneic	CP/Flu	30 to 300 × 10^6^ cells	NA	NA	NA	NA	shRNA-based elimination of TCR
NCT03940833	Asclepius Technology Company Group (Suzhou) Co., Ltd	BCMA CAR-NK 92	Human	1/2	20 (estimated)	BCMA (CAR NK)	NA	NA	NA	Allogeneic	NA	NA	NA	NA	NA	NA	NA
NCT04244656	CRISPR Therapeutics AG	CTX-120	NA	1	80 (estimated)	BCMA	NA	NA	Ex vivo using CRISPR-Cas9	Allogeneic	NA	NA	NA	NA	NA	NA	NA
NCT04171843 (PBCAR269A-01)	Precision BioSciences, Inc.	PBCAR269A	NA	1/2a	48 (estimated)	BCMA	NA	NA	NA	Allogeneic	CP/Flu	0.6, 2, and 6 × 10^6^	NA	NA	NA	NA	Developed by using the unique ARCUS genome editing technology to modify the cells via a single-step engineering process. By inserting the CAR gene at the T-cell receptor (TCR) locus, this process knocks in the CAR while knocking out the TCR

*EMD* Extra-medullary disease, *MRD* Minimal Residual Disease, *DOR* Duration of Response, *CP* Cyclophosphamide, *Flu* Fludarabine, *CRS* Cytokine Release Syndrome, *PFS* Progression-free survival, *OS* Overall Survival, *ORR* Overall Response Rate, *sCR* Stringent Complete Response, *CR* Complete Response, *VGPR* Very Good Partial Response, *PR* Partial Response.

Some other noteworthy BCMA-CAR T products are the human-derived and fully humanized CARs (JCARH125, MCARH171, and FCARH143), designed to reduce the graft-versus-host disease (GVHD) and to prolong the persistence of the T cells^47^. ORRs to these treatments were a remarkable 64, 100, and 82%, respectively. The efficacy of JCARH125, in particular, was reported to be associated with T-cell product that is of high CD3+ purity and enrichment of early-memory phenotype and polyfunctionality^48^.

Described in Table 2 are many more BMCA CAR T trials that are ongoing and recruiting, with different products demonstrating different levels of efficacy. A recent meta-analysis study on a total of 23 different BCMA-CAR T-cell products that have been used in a total of 640 patients, reported an average ORR of 80.5%, with 44.8% CR and 12.2 months median PFS^49^.

## Limitations to BCMA-CAR-T therapy

Alas, the promising results from anti-BCMA CAR T-cell therapies do not come without its own set of challenges.

### Therapeutic resistance

The underlying mechanisms for therapeutic resistance have remain largely unclear, but tumor heterogeneity, and antigen escape have been implicated^50^.

Heterogeneously expressed BMCA at the intra-tumor level can lead to preferential targeting of cells with high BCMA while sparing those with low/zero BCMA expression, resulting in the outgrowth of the latter clones^33,34,47^. Indeed, BCMA often loses its expression upon disease relapse after the first CAR T infusion, suggesting the selection for BCMA−negative MM clones by the CAR T cells^38,51–53^.

For BCMA antigen escape, one of the most well-described ways is the erroneous physiology of the BCMA antigen. BCMA can be inadvertently transferred from tumors to T cells in a process called trogocytosis, causing T-cell fatricide^3,50^ or it can be shed into the blood circulation (now called serum BCMA (sBCMA)), mediated by γ-secretase^47^. Both can cause dampening of tumor cell recognition^38,54^. Although lower sBCMA level is indeed associated with good ORR^36,38,55,56^, it does not always correlate with CAR T dose–response and its expression remains low at the time of relapse with increase disease burden^52^, indicating that mechanism of resistance can extend beyond tumor intrinsic factors.

In relation to this, molecular BCMA aberration was recently highlighted by a study using longitudinal single-cell transcriptomic and bulk genomic analysis. They identified that resistance to bb2121 in a patient was associated with biallelic loss of BCMA. Majority of the MM cells demonstrated heterozygous deletion of chr16p, containing the BCMA locus, concomitantly with nonsense mutation of the other allele, rendering a biallelic inactivation of BCMA^52^. This was corroborated by another study which revealed an association of genomic instability with biallelic deletion of BCMA loci upon CAR T-cell infusion^51^. Both alleles were intact before CAR T treatment, therefore, suggesting the occurrence of branching evolution over treatment course and acquisition of genomic aberrancy by MM cells in selecting for BMCA-negative cells.

### Toxicity

Toxicity such as cytokine release syndrome (CRS) and neurotoxicity, mediated by pro-inflammatory cytokines, upon CAR T-cell activation, is another persistent problem. CRS manifests as fever, nausea, and flu in mild cases, but could escalate to hypotension, cardiac arrest, and liver failure in severe cases. On the other hand, neurotoxicity could range from mild confusion and delirium, to severe obtundation, seizure, and white matter degradation^20^. Despite being a plasma cell marker, BCMA is also co-expressed on normal B-lymphocytes, therefore, BCMA CAR T-cell therapy could also introduce on-target/off-tumor effects, where the common manifestations include B-cell aplasia, neutropenia, and immunosuppression that leads to increased infection risks. In the same aforementioned meta-analysis study, an average CRS and neurotoxicity prevalence of 80.3% and 10.5%, respectively, were reported^49^. While toxicities are frequent events, toxicity-related lethality in the patients are controllable. High-grade toxicities are more often observed in patients with heavy tumor burden or on high CAR T-cell dosage^38^. Toxicities are typically managed with readily available drugs such as IL6R-antagonist (tocilizumab) for CRS, and corticosteroids for neurologic symptoms^20^.

## Non-BCMA CAR T cells

The emergence of next breakthrough molecule is perpetually anticipated. Various surface antigens have been explored in this pursuit. This section documents the progress made by some of the more well-studied myeloma antigens (Table 3).

**Table 3:** Non-anti-BCMA CAR T-cell clinical trials in multiple myeloma (true to the time of writing).

Trial name	Sponsor company	ScFV origin	Phase	*n*	Antigen	CAR name	Co-signaling domain/ CD3z	Transfer method	Cell source	Conditioning	Dosage	Responses
NCT02135406	University of Pennsylvania	Murine	1	10	CD19	CTL019/tisagenlecleucel	4-1BB	Lentiviral	Autologous	CP/Flu	1.1–6.0 × 10^8^	VGPR: 6; PR: 2; PD: 2
NCT01886976	Chinese PLA General Hospital	Murine	1/2	5	CD138	CART-138	4-1BB	Retroviral	Autologous	CP/Flu	0.44–1.51 × 10^7^	SD: 4; PD: 1
NCT00881920	Baylor College of Medicine	Murine	1	16 (7MM)	κ light chain	κ.CARTs	CD28	Retroviral	Autologous	CP	2.0 × 10^8^	4 SD of 7 MM
NA	European Commission and CARAMBA	NA	1/2	25	SLAMF7	NA	NA + EGFRt	Sleeping beauty	Autologous	NA	NA	NA
NCT03958656	National Cancer Institute (NCI)	NA	1	42	SLAMF7	NA	CD28 or 4-1BB/CD3z + inducible caspase 9 (IC9) cell-suicide	NA	Autologous	CP/Flu/Rimiducid	0.3– 12.0 × 10^6^	NA
NCT04142619	Cellectis S.A.	NA	1	18	SLAMF7 (TALEN-targeted gene editing TCR and SLAMF7)	UCARTCS1	4-1BB	NA	Allologous	NA	NA	NA
NCT02203825	Celyad	Human	1	12	NKG2D ligands	NKG2D-CAR	DAP10	Retroviral	Autologous	No lymphdepletion	1–3 × 10^7^	NA
NCT03464916	Sorrento Therapeutics, Inc.	NA	1	72	CD38	CAR2 Anti-CD38 A2	NA	NA	Autologous	NA	NA	NA
NCT03125577	Shenzhen Geno-Immune Medical Institute	NA	1	100	CD38 + CD19	Combination of CD19 with various other CDs in heme-malignancies	CD28/CD137/CD27 + inducible Casp9	Lentiviral	Autologous	Patient dependent	Patient and conditioning agent-dependent	NA
EU-CART	EU Horizon 2020 program	NA	1/2	NA	CD44v6 (+HSV-TK suicide gene)	NA	CD28	Retroviral	Autologous	NA	NA	NA
NCT01716364	Peter MacCallum Cancer Centre, Australia	NA	1	6	Lewis Y	LeY	CD28	Retroviral	Autologous	NA	NA	NA
NCT03638206	Shenzhen BinDeBio Ltd.	NA	1/2	73	NY-ESO-1	NA	NA	NA	Autologous	CP/Flu	NA	NA

### SLAMF7/CS1

The attractiveness of employing signaling lymphocytic activation molecule F7 (SLAMF7) for CAR targeting stems from elotuzumab, the first SLAMF7 humanized moAb that was FDA-approved for RRMM^57,58^. Anti-SLAMF7 CAR T cells deriving from elotuzumab showed preclinical evidence of rapid cytolysis in primary myeloma samples and elimination of extramedullary cells in xenografts^59^. SLAMF7-CAR T cells incorporating on/off suicide gene for enhanced safety and tolerability was later developed for clinical investigations (NCT03958656 and NCT03710421). A European group has also engineered a novel SLAMF7 CAR T-cell model utilizing non-viral gene transfer approach of the Sleeping Beauty transposon system. Their proprietary product is currently being tested in Phase I/II CARAMBA trial (https://www.caramba-cart.eu/patients/)^60^.

Signifying a milestone, the allogenic anti-SLAMF7-CAR T cell (UCARTCS1) became the first ‘off-the-shelf’ CAR T-cell product in MM gaining FDA-approval for clinical trial (MELANI-01). UCARTCS1 was manufactured using healthy allogenic T cells with the TALEN gene editing technology to eliminate endogenous TCR and SLAMF7 expressions, intended to reduce the risk of GVHD and T-cell fratricide^61^.

Importantly, a novel anti-SLAMF7/BCMA bispecific CAR T-cell product is under preclinical development with aim of increasing tumor coverage and overcoming antigen loss. These CAR T cells with single chain antibody containing two ligand-binding domains (one for SLAMF7 and another for BCMA) connected in tandem, showed enhanced activity, compared to T cells expressing individual CARs. In combination with PD1 checkpoint inhibitor, it confers accelerated tumor clearance in vivo^62^.

### CD38

The clinical success of anti-CD38 moAb (daratumumab and the second-generation isatuximab) in MM^63–65^, underlies the development of anti-CD38 CAR T cells. Preclinical evidence of effective anti-myeloma effects of anti-CD38 CAR T-cell therapy^66,67^ has led to the initiation of various clinical trials, one of which investigates anti-CD38 CAR T cells as monotherapy in RRMM (NCT03464916), while others explore its combination with different CAR T cells, including anti-BCMA, anti-CD19, anti-CD138, anti-CD56, and anti-NY-ESO-1^3^. A dual-specificity anti-CD38/BCMA CAR T-cell product is being evaluated (NCT03767751).

It is noteworthy, however, that CD38 is generally not highly expressed on myeloma cells and its expression can be downregulated in advanced disease;^67,68^ thus, resistance to the anti-CD38-CAR-T may be expected. There is also a likelihood for on-target/off-tumor toxicity as CD38 is also expressed on activated T cells (thereby, increasing the risk of T-cell fratricide), NK cells and normal prostate, neuronal, and muscle cells.

### CD19

Although CD19 is typically absent in matured plasma cells, minor subsets of myeloma cells with unique propagating properties express low CD19, associated with drug resistance and relapse-promoting properties^3,54^, making it a plausible therapeutic target. A proof-of-concept pilot study was conducted with anti-CD19 CAR T cells, Kymriah. Out of 12 patients treated, six achieved VGPR, two achieved PR, and two other experienced progressive disease (NCT02135406)^13^. Importantly, a cocktail CAR T product of anti-CD19/anti-BCMA produced an impressive 100% ORR (NCT03455972). Despite more than 1000-fold cell expansion at the peak data-point, patients experienced only mild (Grade 1–2) CRS and no neurotoxicity.

### TACI

Transmembrane activator and CAML interactor (TACI), like BCMA, is a member of the TNF superfamily^34^. Both TACI and BCMA share the same activating ligands, APRIL and BAFF, which when bound to their receptors, confer MM growth and survival. Based on this biological nature, ligand-based CAR T cells directed against APRIL have been developed to concurrently target both the TACI and BCMA signaling pathways^69,70^. In an in vivo model, APRIL-directed CAR T cells eradicated BCMA^+^TACI^−^ and BCMA^−^TACI^+^ tumors, but monospecific anti-BCMA CAR T cells failed to contain the proliferation of the BCMA^−^ cells, suggesting the potential application of APRIL-directed CAR T cells in cases associated with BCMA loss^69^. More recently, another APRIL-targeting CAR T-cell product with a refined molecular design has been reported^70^. This trimeric APRIL-based CAR T cells have enhanced binding to BCMA and TACI receptors with increased cytolytic activities, compared to its monomeric counterpart. Despite these promising preclinical data, the only clinical trial on APRIL-targeting CAR T cells conducted till date was terminated due to insufficient efficacy (NCT03287804).

There is only one reported CAR T-cell product in development that specifically targets TACI surface antigen. It is the bispecific TACI/BCMA CAR T cells, that possess efficient in vitro and in vivo cytotoxicity towards MM cells^71^.

Besides myeloma cells, TACI is also found on the immunosuppressive regulatory T cells (Tregs). Targeting TACI, thus, may not only cause cytotoxicity through direct cytolysis, but also by indirectly manipulating the hostile microenvironment imposed by Tregs^34,72^, rendering a good two-pronged approach.

### CD138

CD138 is a highly expressed surface molecule on MM cells, used clinically as a selection marker for plasma cells. The Chinese PLA Hospital tested a second-generation 4-1BB/CD3 CD138-targeted CAR T-cell product as a monotherapy in MM; however, only modest therapeutic response was reported (NCT01886976). This data was obtained from a small sample size (*n* = 5), thus results may not be sufficiently powered for meaningful interpretation. Anti-CD138 CAR T cells have also been incorporated into other CAR T-cell cocktail (BCMA, CD38, CD19, SLAMF7) trials but no substantial data can be obtained (NCT03778346 and NCT03196414).

Despite its overexpression in myeloma cells, it should be noted that CD138 is present in other normal tissues such as epithelial, endothelial, and vascular smooth muscle cells, again, underlining the possibility of on-target/off-tumor effects. This drawback was evident in past clinical trials investigating the anti-CD138 antibody-drug conjugate for MM treatment where some patients reported serious mucosal and skin toxicity^13^.

### Others

Amidst the intensive research on CAR T-cell therapy, various other target antigens have also been examined, some of the worth mentioning ones are G-protein-coupled Receptor Class C Group 5 Member D (GPRC5D), CD56, Lewis Y, CD44v6, and Kappa-light chain. These molecules were studied because of their surface expression and prognostic implications^66^. Comprehensive data from trials of these CAR T-cell products are mostly unavailable for evaluation but for some that have trial data released, there is not much excitement as the patients reported modest to low responsiveness^54^. Various other molecules, such as CD229, integrin β7, CD70, and CD126 (IL6R) involved in plasma cell biology are being investigated preclinically^3,73^.

## Technological advances of CAR T-cell manufacturing in myeloma

As CAR T-cell therapies continue to evolve, multi-modal engineering of effector cells offers the prospect of tackling increasingly complex disease settings. To address the issue of antigen escape upon disease relapse, CARs targeting double epitopes of BCMA have been explored. LCAR-B38BM and JNJ-4528 were built to express two BCMA-binding domains to increase its binding affinity to low BCMA-expressing myeloma cells. The caveat to this approach, however, is that there is yet a conclusive answer as to whether a certain threshold of BCMA expression is required for optimal tumor recognition and cytotoxicity. The study by University of Pennsylvania (NCT02546167) revealed no correlation between baseline BCMA expression and the response rate;^74^ in contrast, the FCARH143 trial (NCT03338972) found differential BCMA expression on tumor cells of long-term responders versus relapse patients prior to the treatment. In the many BCMA clinical trials conducted till date, the baseline BCMA is or is not an inclusion criterion and if it is, the range encompasses a mere observable expression (~1%) to a high >50% (Table 2). This anticipated inter-study heterogeneity makes it difficult to evaluate the potential factors contributing to safety and efficacy. A single clinical trial that sets a range of different baseline levels may be able to inform on the antigen threshold required for desired anti-myeloma effect and if a lower baseline BCMA confers more rapid resistance/relapse.

In overcoming clonal selection for single antigen loss, dual/multi-antigen targeting has been approached by either (a) infusing patients with multiple CAR T-cell products in a cocktail regimen or (b) having double ScFVs for different antigens within one T cell. Some of the known antigens that are being co-expressed with BCMA CAR are SLAMF7, CD19, and CD38 (Table 2). This strategy is pertinent considering these targets have remained stably expressed at progression in BCMA-negative setting^51^. However, it is crucial to consider the accompanying challenges. For example, in (b), controlling the ratio of positivity of each CAR within the T cells may be technically challenging. The difficulty to define the expression level of either of these CARs on a single T cell would create a highly heterogenous pool of CAR T cells that may impinge on its tumor selectivity and ultimate potency. Furthermore, as with single-targeting CARs, the dual/multi-targeting CARs can also elicit selective pressure that could be stronger due to enhanced immune response, resulting in simultaneous escape of both antigens. Beyond technical complexities, another apparent challenge is the risk of high-grade CRS that comes with augmented T-cell activation from concurrent antigen targeting^75^.

To ameliorate the toxicities arising from hyperactivated CAR T cells and on-target/off-tumor specificity, some CARs have been modified to contain an inhibitory/suicide gene, which when required, can be triggered to switch off T-cell signaling to avert ensuing toxicity. Suicide genes such as truncated EGFR (NCT03070327 and NCT03093168), iCasp9 (NCT03958656 and NCT03125577), and HSV-TK (NCT04097301) are currently being investigated in myeloma. Other safety strategies such as bispecific T-cell engager and tandem CAR have yet to be explored in myeloma and are therefore worth looking into^20^.

Thus far, generic CAR models consist of either CD28 or 4-1BB as co-stimulatory domain. The choice between these two molecules for CARs is essential as it determines the rapidity of immune activation and the persistence of the CAR T cells. CD28-harboring cells were shown to possess higher potency, rapid expansion, and ability to elicit quick cytokine surge following antigen stimulation, whereas, 4-1BB-containing CARs demonstrated lower signaling intensity, gradual activation, and a memory stem cell-like phenotype^76^. Although the former is associated with swift and robust cytolytic activities, its brisk signaling kinetics was associated with faster T-cell exhaustion, thus, resulting in less durable responses. They lack ongoing persistence compared to their 4-1BB counterpart, making them a likely inferior choice for a CAR construct^13,66^. The lesser preference for CD28-CAR in MM is evident (Table 2), whereby, after the first-in-human CD28-equipped BCMA-CAR T trial (NCI), subsequent CAR T products predominantly carry 4-1BB.

Second-generation CARs are leading the pack in CAR T-cell research and investigation of the third- and fourth-generation CARs in myeloma is scarce. Although the third-generation CARs should logically possess superior performance than its predecessor, most clinical data, at least in B-cell malignancies, demonstrated minimally augmented efficacy^77^. In MM, the efficacy of the third-generation BCMA- and CD19-CAR constructs (CD28-OX40-CD3ζ) were tested by infusing the product cocktail into RRMM patients, post-ASCT. The ORR was 100%, with low grade CRS^54^. This then begs the question of whether the response was truly attributable to the activity of third-generation CARs or was it an effect of dual-antigen targeting with successful ASCT. A study making a parallel comparison between the second- and third-generation CARs in a single experimental setup may be imperative. Likewise, the concept of fourth-generation CARs is well-received for its role in immunomodulation and reprogramming of tumor microenvironment; however, the knowledge on its efficacy compared to second- and third-generation CARs is limited by the lack of clinical pursue^28^. A search on the ClinicalTrial.gov registry’s portal revealed 16 trials on fourth-generation CARs, two of which are for myeloma. These trials explore the efficacy of single or multi-antigen CAR T-cell compounds (BCMA, CD138, CD38, Integrin β7) (NCT03778346 and NCT03271632). While novel designs intended to increase CAR T-cell efficacy in myeloma are crucial, practical elements should also be considered, as higher innovations are often more labor-intensive and less cost-effective, not to mention the accompanying toxicity profile^28^.

Short clinical remissions in MM have been attributed to low persistence of the BCMA CAR-T cells. To circumvent this, bb21217 that was derived from its precursor bb2121, was cultured ex vivo in the presence of PI3K inhibitor bb007, to enrich T cells with memory-like phenotype. The benefit of this approach was proven, when bb21217, but not bb2121, induced tumor clearance during a second tumor challenge within the same mice, without the need for re-administration of the product^78^. Bbb21217 displayed higher levels of CCR7 and CD27 compared to bb2121, suggesting higher levels of memory-like T cells, and lower levels of CD57, a marker of T-cell exhaustion. The latest update on bb21217 indicated 55% ORR with the CAR T cells being detectable up to 18 months^39,43^. Additionally, the contribution of immunosuppressive factors in tumor microenvironment to the lack of durability of CAR-T cells is also notable. A new fully human BCMA-CAR harboring a dominant negative domain against the immunosuppressive TGF-β was recently reported to exhibit robust proliferation and persistence, and remains functionally impervious to hostile TGF-β-rich microenvironment^79^.

Another manufacturing optimization that has also been attempted to amplify the potency of CAR T cells in myeloma is the T-cell composition. A higher CD4:CD8 T-cell ratio utilized at the pre-transduction and expansion stage was associated with greater in vivo BCMA-targeted CAR T-cell expansion and response^74^, corroborating the reports in the CD19-targeted CAR T-cell therapy in lymphoma and ALL^80,81^. JCARH125 was infused into the RRMM patients (EVOLVE) with a designated CD4:CD8 ratio of 1:1. Latest clinical data reported robust expansion of these products at all dose levels, with patients’ ORR (82%) closely associated with the composite T cells^82^.

Gene delivery method is another variable in MM CAR T manufacturing, of which virus transduction is most conventionally opted. Given that this system comes with the risk of random insertional mutagenesis and is often associated with laborious and costly manufacturing procedures, other non-viral ways of introducing CARs into T cells have been introduced. The first non-viral CAR T-cell product for myeloma was developed by Poseida Therapeutics utilizing the piggyback-transposon technology (P-BCMA-101). This mRNA electroporation method demonstrated >95% efficiency and a more stable and higher CAR expression, coupled with a prolonged activity from the expansion of T cells with memory stem cell phenotype, when compared to those delivered by virus^74,83,84^. Additionally, another non-viral system, the Sleeping Beauty, was used to introduce SLAMF7-CAR into the effector cells and has demonstrated good integration^60^.

Another CAR T manufacturing consideration is the origin of the moAb. One of the described therapeutic limitations of the otherwise successful CD19-targeted CAR T cells in lymphoma was T-cell fratricide due to host immune anti-murine CAR responses^80^. Integrating the less immunogenic humanized ScFVs into T cells is therefore a pragmatic approach. Indeed, the fully humanized BCMA-targeted CAR T cells (FCARH143 and JCARH125) developed by MSKCC exhibited rapid expansion, eradication of large tumor burden, and more durable patient response^66,78^. A novel, fully human, heavy-chain-only anti-BCMA binding domain (FHVH33) instead of a full ScFV, that was engineered to avert host immune cells’ recognition, has been shown to induce deep and durable responses with manageable toxicities^53^.

## Perspectives

The quest to identify novel targetable myeloma antigens is crucial; however, those that have been deliberated thus far did not seem able to uphold the potential of BCMA’s. As a single agent, non-BCMA CAR T cells lacked meaningful outcomes. Refinements to these CARS such as rendering them bispecific often requires BCMA being the accompanying targeted antigen. With the impending FDA-approval of bb2121, studies on elucidating the underlying resistance mechanisms and ways of circumventing it, is indispensable.

Synthetic biology is crucial for engineering more potent BCMA-targeting CAR T cells, nevertheless, maintaining tumor-associated-BCMA expression and preventing antigen loss, is required for their execution of potency. In this instance, pharmacological means have been explored. γ-secretase inhibitor (GSI) treatment was able to increase BCMA density on tumor surface alongside decreased sBCMA concentration and improved CAR T-cell anti-tumor activity^85,86^. Oral administration of GSI with CAR T cells is currently being investigated in a Phase I trial (NCT03502577). Out of 10 evaluable patients, 30 and 50% achieved sCR/CR and VGPR, respectively^85,87^. Interestingly, all-trans retinoic acid (ATRA), that was reported to rescue daratumumab sensitivity by increasing CD38 expression^88^, was also found to be able to augment BCMA expression through an epigenetic modulation and enhances its recognition by BCMA-CAR T cells^86^. Combination treatment with ATRA and GSI led to higher BCMA density on MM cells, resulting in maximized reactivity. It is, again, imperative that we first clarify the inconclusive notion on the importance of baseline BCMA in conferring responsiveness. Not only is it necessary to ascertain the percentage of plasma cells having BCMA positivity, knowledge on the antigen density in each of the BCMA^+^ cells is also critical for discerning the BCMA threshold responsible for efficient CAR T-cell recognition.

**Fig. 5:**
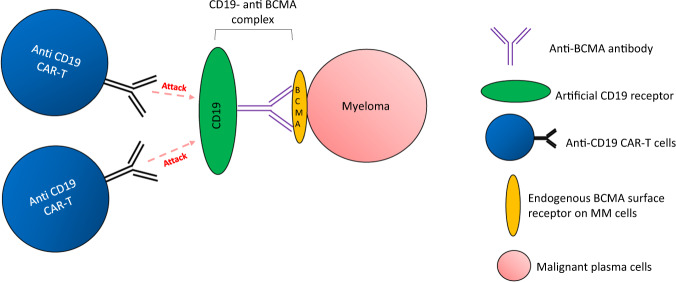
CD19-antigen decoy for innovative targeting of myeloma cells. Increased expression of CD19 is induced on the myeloma cells by artificially creating the protein-anti-BCMA antibody complex that binds to endogenous surface BCMA. Highly potent and specific anti-CD19 CAR-T can be used to target these synthetic myeloma cells.

More recently, new attempts to leverage on the success of anti-CD19 CAR T therapies have emerged. Novel CD19-fusion protein models were developed to reactivate the targetability of relapsed NHL with low CD19 expression^89^ and HER2-positive solid tumors^90^. Effective CD19-specific CAR T-cell-mediated cytotoxicity was observed in cases where artificial CD19 expression was successfully created on the surface of these CD19-low/negative tumor cells. Such innovative strategy may also be considered for MM (Fig. 5). In this instance, a decoy CD19 antigen can be engineered on the surface of myeloma cells by building a CD19-BCMA (or any other specific MM antigen) antibody complex, with the BCMA arm attaching to the tumor antigen. Overexpression of synthetic CD19 on MM would enable us to leverage on the already commercialized CD19-targeted CAR T cells (Kymriah or Yescarta) that are proven highly specific and potent.

Current standard-of-care has proven that combination of multiple agents targeting different mechanisms is the more effective way for disease management. However, investigations into BCMA CAR T therapy in combination with the diverse portfolio of anti-myeloma agents are rather limited. A preclinical work combining CAR T cells with lenalidomide found that the latter could enhance the persistence and activity of the former^91,92^. Another study reported that BCMA inhibition by the novel BCMA-targeting ADC (MEDI2228) could synergize with bortezomib in drug-resistant MM cells^93^. Various signaling inhibitors such as BRD4i and MEKi were shown to boost CAR T cell’s functionality by hampering their immunosuppressive effects but these have never been studied in myeloma^94,95^. Plausibly in a drug combination study, one could investigate whether or not to incorporate an agent concomitantly with the CAR T-cell infusion or for maintenance post-CAR T therapy, which may be useful for patients with huge MM burden.

The impressive results obtained thus far from CAR T-cell therapy trials in advanced and heavily pretreated MM patients have prompted the question of whether CAR T cells should be used as an earlier-line therapy. The lower disease burden in newly diagnosed or early-stage diseases may represent higher chance of achieving tumor clearance. In these cases, CARs with CD28, instead of 4-1BB co-stimulatory domain, may be a better choice as they elicit faster immune response. Swift activity of CD28-equipped-CAR T cells would be useful for prompt therapeutic effects, thereby, better controlling the disease at an early stage, preventing progression. This would appear to be even more critical for high-risk patients such as double-hit or functional high-risk myeloma whose disease often advances quickly even with modern treatment strategy^5^. Attempt into investigating bb2121 in high-risk newly diagnosed patients has been initiated in KarMMa-4 trial (NCT04196491)^96^.

Additionally, myeloma has long been associated with quantitative and functional deficient T cells, particularly in the progressive, refractory disease. Acquisition of T cells from healthy donors is an attractive solution to the problem of insufficient and inferior CAR T cells. There are a few known allogenic compounds in MM that have gained IND clearance from the FDA, namely, UCARTCS1 (SLAMF7), CTX120 (BCMA), ALLO-715 (BCMA), CYAD-211 (BCMA), and PBCAR269A (BCMA)^97,98^. These products were conceived using different technologies including the non-gene editing method to eliminate TCR (CYAD-211) and single-step platform to concurrently knock-out TCR while knocking-in CAR (PBCAR269A)^99^. Another BCMA allogeneic compound (ALLO-605) that is being evaluated pre-clinically incorporates additional chimeric features to enhance cytokine signaling^100^. Besides addressing the T-cell quality, off-the-shelf accessibility of these products also means that we can by-pass the long and complex autologous manufacturing process, thus, allowing for more rapid frontline therapies. The sustained availability of these allogenic CAR T cells will be useful for eradication of residual malignant cells, without the need for multiple rounds of painstaking autologous T-cell harvesting from the patients. In short, a cell bank with universal CAR T cells for allogeneic cell transfer would provide more flexibility in the application, faster delivery to the patients with less cost-intensive manufacturing.

Of late, CAR NK in myeloma is slowly gaining traction, owing to its better safety profile, versatility in its mechanisms of killing, and lower risk of GVHD, thereby higher feasibility for allogenic manufacturing that translates into cost effectiveness^101^. Several preclinical studies have demonstrated cytotoxic activity and MM growth inhibition using CAR NK cells against various targets, including CS1, CD138, BCMA, and NKG2D ligands^102^. There is currently one ongoing BCMA-CAR NK Phase 1/2 study in RRMM (NCT03940833). Furthermore, leveraging on the stem cells reprogramming system, an innovative CAR NK model, harboring a recombinant IL-15 signaling complex, was generated from induced pluripotent stem cells (iPSC). It has shown therapeutic efficacy alongside good synergism with moAb drugs (daratumumab, elotuzumab, and anti-CD19)^103^. With the reported safety properties of CAR NK cells over CAR T cells, we envision that the CAR NK therapy research field in MM will gain an incremental interest that will allow for the diversification of MM cell therapy product portfolio, although its potential drawbacks are also to be cautiously weighed.

On top of bio-engineering and diverse clinical testings, basic mechanistic research is also of importance as it provides insights into the fundamentals of immune response. Understanding the basic biology of T cells would enable us to produce ‘fitter’ CAR T cells that possess enhanced proliferation and longer endurance. Edging on the advancement of CRISPR-Cas9 technology, a genome-wide screening process can be performed to identify novel genes such when manipulated, could confer enhanced resilience and capabilities to the CAR T cells. Epigenetic mechanisms such as chromatin accessibility, DNA methylation states, RNA landscape in T cells, immune profiling, and identification of immune checkpoint signature are some of the key areas for further investigation. Recoding the T-cell behavior via gene knock in/out approach would be helpful in addressing the issue of innate T-cell defects and inefficient pool of effector cells. To understand disease recurrence in a highly heterogeneous pool of MM cells, leveraging on the advent of single-cell sequencing technology may be able to help us obtain a high-level understanding of the temporal evolution on genetic and epigenetic aspects of the immune cells. A recent single-cell study of a plasma cell leukemia patient revealed distinct CAR T-cell subsets having different expression, proliferation, and cytotoxicity, indicating stage-specific changes along the developmental trajectory^104^. Considering this and MM heterogeneity, it would therefore be interesting to observe how different genomics and transcriptomics, such as different TC classes and RNA editing signatures, could dictate expression of surface targets that can be of use for immune targeting.

Beyond the basic biology, ex vivo and scaling processes also play a big part in CAR T-cell production^37^. Logistic factor involving time elapses between apheresis and delivery of manufactured CAR T cells back to patients is critical as the current turnaround time of 2–4 weeks renders CAR T-cell therapy unsuitable for patients with rapidly progressing disease. One emerging technology addressing this lengthy manufacturing issue is GraCell Bio’s propriety FasT CAR platform, in which product release can be achieved within 22–36 h using an expedited protocol to activate and transduce T cells without the need for further ex vivo expansion. A dual BCMA-CD19 CAR T therapy was developed using this platform and the first-in-human study has demonstrated an effective 93.8% ORR^105^. Such finding should be a precursor to further identification of key bottlenecks along the whole CAR T-cell manufacturing pipeline (Fig. 4) and ways to optimize the steps to allow for less costly and cumbersome processes.

## Conclusion

The CAR T-cell field in MM has indeed come a long way since the first BCMA-targeted CAR was developed in 2013. In our constant pursuit to develop personalized medicine, CAR T-cell therapy is perhaps the ultimate, as nothing can be more personalized than a treatment that harnesses the patient’s own immune system for tumor destruction. BCMA has gained the ‘holy grail’ recognition for the CAR T-cell field in myeloma and as long as continuous effort is invested into circumventing the barriers that have limited the success of current BCMA-directed CAR T-cell products, we may soon be able to produce an optimized product for MM that could be within the same realm of success as CD19-targeting CARs in lymphomas and ALL. Although the emergence of novel drugs has brought about significant improvement in patients’ outcome, these synthetic agents are physiologically eliminated from the body over time, making it difficult to produce a durable response without repetitive administration. CAR T cells, in constrast, is deemed a ‘living drug’ as they would be induced as long as targeted tumor antigen exists, thus making its therapeutic effect sustainable. If its efficacy is long-standing enough, we may well be on our way to having patients with lasting remission. With this and the anticipation for FDA’s approval of bb2121 this year, we have every single reason to remain hopeful and optimistic that CAR T-cell therapies may one day render MM a chronic but highly manageable and curable disease.
